# *Notes from the Field*: Expanding Birthing Hospital Enrollment in the Vaccines for Children Program to Increase Infant Immunization Against Respiratory Syncytial Virus — United States, October 2023–March 2025

**DOI:** 10.15585/mmwr.mm7437a2

**Published:** 2025-11-20

**Authors:** Kerry E. Olmsted, Terrin Ramsey-Omonua, Ebony S. Thomas, James T. Lee, Llandess Owens, Sam Graitcer, Jamie Mells

**Affiliations:** ^1^Immunization Services Division, National Center for Immunization and Respiratory Diseases, CDC; ^2^VAAS Professionals, LLC, Atlanta, Georgia.

**Summary**

**What is already known about this topic?**

Nirsevimab, a long-acting monoclonal antibody that protects infants against respiratory syncytial virus (RSV), should be administered within 1 week after birth to infants not protected by maternal RSV vaccination. The Vaccines for Children (VFC) program provides CDC-purchased immunizations, including nirsevimab, to eligible children at no cost.

**What is added by this report?**

A CDC effort with professional organizations and health departments to enroll birthing hospitals in VFC was associated with an increase in enrolled birthing hospitals from 763 (27.1% of 2,817 facilities) at the beginning of the 2023–24 RSV season to 1,021 (36.2%) by the end of the 2024–25 RSV season. The number of nirsevimab doses ordered approximately doubled.

**What are the implications for public health practice?**

Continued efforts to increase birthing hospital VFC enrollment can support expanded access to RSV immunization.

Respiratory syncytial virus (RSV), the leading cause of hospitalization among U.S. infants, results in 50,000–80,000 associated hospitalizations and 100–300 deaths among children aged <5 years each year (*1*). In 2023, the Advisory Committee on Immunization Practices (ACIP) recommended two options for preventing severe RSV in infants: maternal RSV vaccination during pregnancy (*2*) or administration of nirsevimab, a long-acting monoclonal antibody to infants (*1*). Nirsevimab is recommended for infants aged <8 months during their first RSV season (October–March in most of the United States); ideally, it should be administered during the birth hospitalization or within the first week of life. In September 2023, ACIP passed a resolution to add nirsevimab to the Vaccines for Children (VFC) Program, a public-private partnership that provides CDC-purchased vaccines to VFC-eligible children (those who are uninsured or underinsured, insured through Medicaid, or who are American Indian or Alaska Native) at no cost. Approximately one half (52.2%) of U.S. children aged 19–35 months are VFC-eligible, and among those, 93.4% are insured by Medicaid (*3*). Medicaid-insured infants have a higher incidence of severe RSV infection than do privately insured infants (*4*). Providers enrolled in the VFC program are able to administer nirsevimab at no cost to eligible children. Enrollment of birthing hospitals in VFC thus has the potential to expand infant immunization against RSV. This report describes enrollment of U.S. birthing hospitals (those with more than one birth during the previous year or at least one registered maternity bed) in the VFC program since the introduction of nirsevimab.

## Investigation and Findings

### Enrollment of Birthing Hospitals in VFC

Birthing hospitals are well positioned to provide timely access to nirsevimab, perhaps especially to Medicaid-insured infants who are less likely to receive a pediatric checkup within the first week of life than privately insured infants (*5*) and who therefore might miss the recommended window for receiving nirsevimab if it is not administered during the birth hospitalization. Enrolling as a VFC provider allows birthing hospitals to offset the high cost of nirsevimab.*

In 2023, CDC undertook efforts to facilitate birthing hospital enrollment in the VFC program. In April 2023, CDC held focus groups with 11 U.S. jurisdictions on facilitators and barriers to enrolling birthing hospitals in VFC. Many jurisdictions reported challenges in developing relationships with birthing hospitals, a need for capacity building within jurisdictions, and a need for flexible VFC policies. In response, to streamline administrative processes for providers, CDC updated VFC policies to facilitate nirsevimab ordering and inventory management by VFC providers and encouraged birthing hospitals to implement VFC policies reducing the required number of stocked immunization products to only those recommended within 1 week of birth (i.e., hepatitis B vaccine and nirsevimab), rather than all recommended childhood immunizations.

Beginning in August 2023, CDC partnered with professional organizations and health departments^†^ to support birthing hospital enrollment in VFC through development and dissemination of educational and training resources and establishment of learning collaboratives that included facilitated discussions for sharing lessons learned and promising practices. U.S. birthing hospital enrollment in VFC and doses of nirsevimab purchased by these facilities were estimated across the two RSV seasons following the recommendation for nirsevimab for infants (October 1, 2023–March 31, 2024 [2023–24] and October 1, 2024–March 31, 2025 [2024–25]).

### Identification of Birthing Hospitals Enrolled in VFC

A list of U.S. birthing hospitals was created using the 2022 American Hospital Association Annual Survey and the 2024 Maternity Practices in Infant Nutrition and Care survey to match birthing hospitals to enrolled VFC hospitals^§^; jurisdictions^¶^ reviewed data for accuracy. This activity was reviewed by CDC, deemed not research, and was conducted consistent with applicable federal law and CDC policy.**

### Birthing Hospital Enrollment During the 2023–24 and 2024–25 RSV Seasons

Among all 2,817 U.S. birthing hospitals, the number enrolled in VFC increased from 763 (27.1%) at the beginning of the 2023–24 RSV season to 1,021 (36.2%) by the end of the 2024–25 season (Table), covering approximately 41.8% of U.S. births in hospitals. The largest increase occurred among jurisdictions in the U.S. Census Bureau Northeast Region, from 158 (44.4%) birthing hospitals to 266 (74.5%). During the 2023–24 season, birthing hospitals ordered 46,738 VFC doses of nirsevimab; during the 2024–25 season, the number of doses ordered more than doubled, to 102,057.

**TABLE:** Summary of change in birthing hospital enrollment in the Vaccines for Children program across two respiratory syncytial virus seasons — United States, October 1, 2023–March 31, 2024, and October 1, 2024–March 31, 2025

Jurisdiction	Birthing hospitals*		
	Total no. at the end of the 2024–25 RSV season	No. (% of total) enrolled in VFC	
		Beginning of the 2023–24 RSV season	End of the 2024–25 RSV season
**United States**	**2,817**	**763 (27.1)**	**1,021 (36.2)**
**State or city**			
Alabama	**49**	7 (14.3)	10 (20.4)
Alaska	**19**	10 (52.6)	11 (57.9)
Arizona	**48**	24 (50.0)	24 (50.0)
Arkansas	**35**	18 (50.0)	20 (57.1)
California	**226**	34 (15.0)	54 (23.9)
Colorado	**63**	6 (9.5)	22 (34.9)
Connecticut	**23**	18 (78.3)	23 (100.0)
Delaware^†^	**7**	2 (28.6)	2 (28.6)
District of Columbia	**6**	0 (—)	1 (16.7)
Florida	**119**	8 (6.7)	13 (10.9)
Georgia	**77**	5 (6.4)	4 (5.2)
Hawaii	**12**	11 (91.7)	10 (83.3)
Idaho	**31**	20 (64.5)	23 (74.2)
Illinois	**74**	2 (2.7)	6 (8.1)
Chicago	**15**	2 (13.3)	4 (26.7)
Indiana	**72**	16 (22.2)	27 (37.5)
Iowa	**53**	21 (39.6)	23 (43.4)
Kansas^†^	**64**	0 (—)	1 (1.6)
Kentucky	**49**	32 (65.3)	34 (69.4)
Louisiana	**52**	14 (26.9)	17 (32.7)
Maine	**19**	9 (45.0)	19 (100.0)
Maryland	**33**	2 (6.1)	6 (18.2)
Massachusetts	**40**	19 (47.5)	31 (77.5)
Michigan	**76**	58 (76.3)	64 (84.2)
Minnesota	**89**	10 (11.2)	11 (12.4)
Mississippi	**46**	17 (37.0)	19 (41.3)
Missouri	**63**	4 (6.3)	5 (7.9)
Montana	**25**	24 (96.0)	23 (92.0)
Nebraska	**55**	0 (—)	0 (—)
Nevada	**20**	8 (38.1)	8 (40.0)
New Hampshire	**15**	7 (46.7)	8 (53.3)
New Jersey	**50**	5 (10.0)	14 (28.0)
New Mexico	**29**	11 (37.9)	11 (37.9)
New York	**78**	2 (2.6)	54 (69.2)
New York City	**38**	26 (68.4)	38 (100.0)
North Carolina	**83**	41 (49.4)	44 (53.0)
North Dakota	**13**	12 (92.3)	5 (38.5)
Ohio	**91**	1 (1.1)	1 (1.1)
Oklahoma	**45**	14 (31.1)	17 (37.8)
Oregon	**47**	2 (4.3)	1 (2.1)
Pennsylvania	**72**	63 (84.0)	60 (83.3)
Philadelphia	**6**	0 (—)	5 (83.3)
Rhode Island	**5**	5 (100.0)	5 (100.0)
South Carolina	**45**	13 (28.9)	14 (31.1)
South Dakota	**23**	13 (54.2)	12 (52.2)
Tennessee	**64**	7 (10.9)	7 (10.9)
Texas	**211**	53 (25.1)	65 (30.8)
Houston	**13**	1 (7.7)	7 (53.8)
San Antonio	**7**	4 (57.1)	4 (57.1)
Utah	**46**	5 (10.9)	24 (52.2)
Vermont	**11**	4 (36.4)	9 (81.8)
Virginia	**58**	7 (11.9)	14 (24.1)
Washington	**54**	23 (42.6)	39 (72.2)
West Virginia	**21**	2 (9.5)	3 (14.3)
Wisconsin	**81**	15 (18.5)	19 (23.5)
Wyoming	**17**	2 (11.1)	1 (5.9)
**U.S. Census Bureau region** ^§^			
Northeast	**357**	158 (44.3)	266 (74.5)
Midwest	**769**	154 (20.0)	178 (23.1)
South	**1,020**	247 (24.2)	301 (29.5)
West	**637**	180 (28.3)	251 (39.4)
**Territory**			
American Samoa	**1**	1 (100.0)	1 (100.0)
Guam	**2**	0 (—)	0 (—)
Northern Mariana Islands	**1**	0 (—)	0 (—)
Puerto Rico	**28**	21 (65.6)	22 (78.6)
U.S. Virgin Islands	**2**	2 (100.0)	2 (100.0)

**Abbreviations:** RSV = respiratory syncytial virus; VFC = Vaccines for Children.

* The total number of known birthing hospitals on March 31, 2025, was used as the denominator for both time points. The denominator did not change meaningfully between seasons.

^†^ Jurisdiction did not provide additional feedback on its birthing hospital enrollment data.

^§^ U.S. Census Bureau regions and divisions of the United States excludes U.S. territories.

## Preliminary Conclusions and Actions

Efforts to enroll birthing hospitals in VFC depend on jurisdiction staff members who administer the VFC program and understand its requirements. Starting July 1, 2025, as a condition of funding support for VFC, CDC requires that each jurisdiction enroll ≥30% of its birthing hospitals in VFC; as of March 31, 2025, 39 of 61 (63.9%) jurisdictions have met this target. Clesrovimab, a new RSV monoclonal antibody, was recommended by ACIP in June 2025 and will be available as an alternative to nirsevimab through the VFC program. CDC continues to partner with jurisdictions and professional organizations to address barriers to VFC enrollment for birthing hospitals and share best practices. Birthing hospital VFC enrollment can support expanded access to RSV immunization among infants at highest risk for severe disease.

## Footnotes

* Before 2023, hepatitis B vaccine, a low-cost immunization, was the only immunization recommended at birth; thus, birthing hospitals had less financial incentive to enroll in VFC. Whereas hepatitis B vaccine costs approximately $15 per dose, nirsevimab costs approximately $415 per dose in the VFC program (CDC Vaccine Price List as of April 1, 2025). Clesrovimab, a new RSV monoclonal antibody product, was recommended by ACIP in June 2025; VFC pricing information is not yet available.

† American Hospital Association, Association of Immunization Managers, America’s Health Insurance Plans, American College of Obstetricians and Gynecologists, American Academy of Pediatrics, American College of Nurse-Midwives, Centers for Medicare & Medicaid Services, and Perinatal Quality Collaboratives.

§ String matching (i.e., searching for a smaller text string within a larger string) using Python (version 3.12; Python Software Foundation) connected birthing hospitals to enrolled VFC providers using hospital names, contact information, and locations. VFC providers were matched to the same list of birthing hospitals at both time points; the total number of birthing hospitals did not change meaningfully from the beginning of the 2023–24 RSV season to the end of the 2024–25 RSV season.

¶ Birthing hospital VFC enrollment is counted by the 61 jurisdictions with VFC funding: all 50 states, the District of Columbia, five U.S. cities (Chicago, Houston, New York City, Philadelphia, and San Antonio), and five U.S. territories (American Samoa, Guam, the Northern Mariana Islands, Puerto Rico, and the U.S. Virgin Islands). VFC is updating its data system to better capture services offered and birthing hospital status.

** 45 C.F.R. part 46.102(l)(2), 21 C.F.R. part 56; 42 U.S.C. Sect. 241(d); 5 U.S.C. Sect. 552a; 44 U.S.C. Sect. 3501 et seq.
